# Supplementary material for: Evasion of antiviral bacterial immunity by phage tRNAs
Source: Nat Commun. 2024 Nov 11;15:9586. doi: 10.1038/s41467-024-53789-y (PMC11555353; doi:10.1038/s41467-024-53789-y)
Supplement: Supplementary file 1 — Supplementary Information [file 41467_2024_53789_MOESM1_ESM.pdf]

Evasion of antiviral bacterial immunity by phage tRNAs

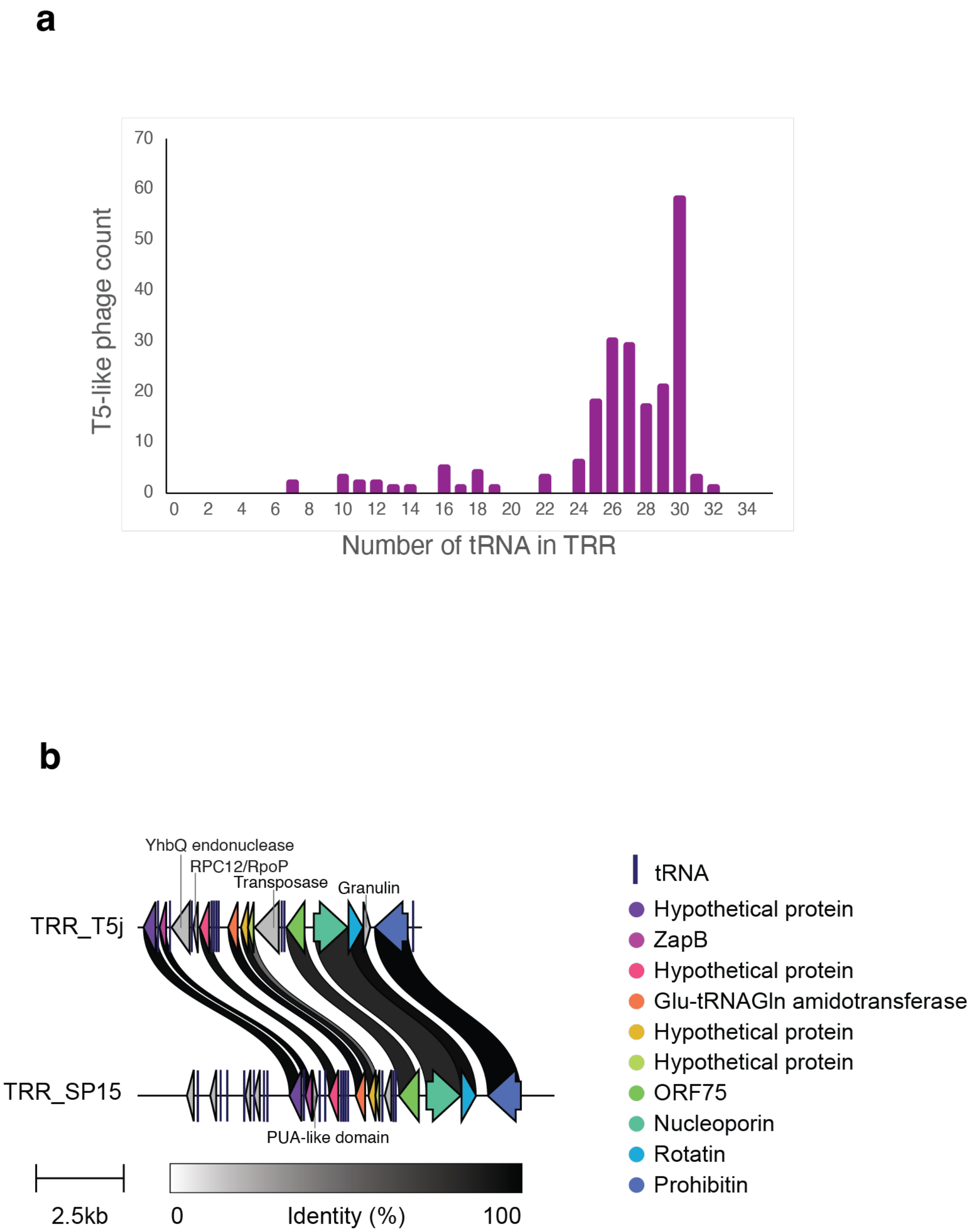

Supplementary Figure 1.

(A) Quantification of tRNA in the tRNA-rich region (TRR) of 203 T5-like phages from the NCBI database.

(B) Genomic alignment of TRR between T5j and SP15. Identical genes shared between T5j and SP15 are highlighted in different colors. The presence of tRNA is indicated by a dark blue vertical line. The alignment was performed using Clinker[1].

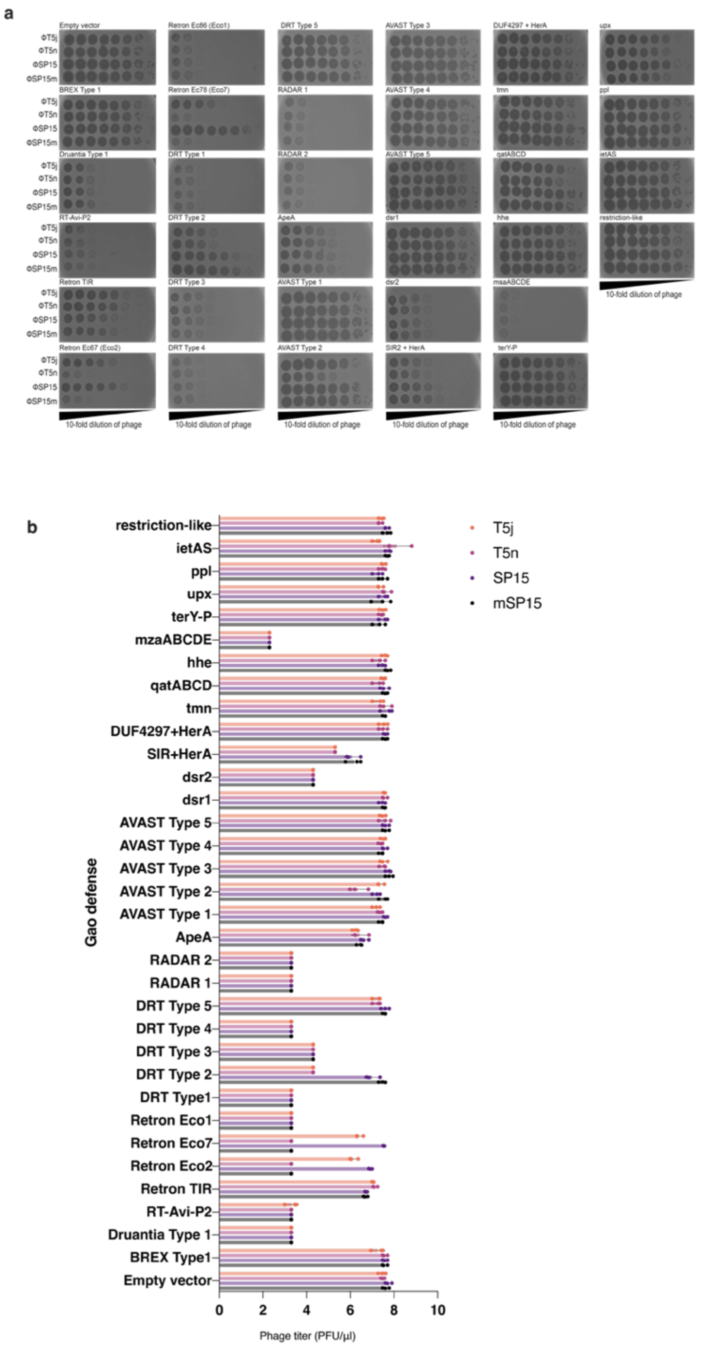

Supplementary Figure 2.

Infectivity of phage (T5j and SP15) and their respective mutants (T5n and SP15m) on bacteria carrying different antiphage defense systems reported in Gao et al[1]. (A) Phage spot assay using 10-fold diluted phage solution. (B) Quantification of phage titer propagated in bacteria carrying the Gao defense system. Experimental results illustrated in (A) and (B) were obtained after performing the experiments in triplicates. Data are presented as mean values ± SD. Source data are provided as a Source Data File.

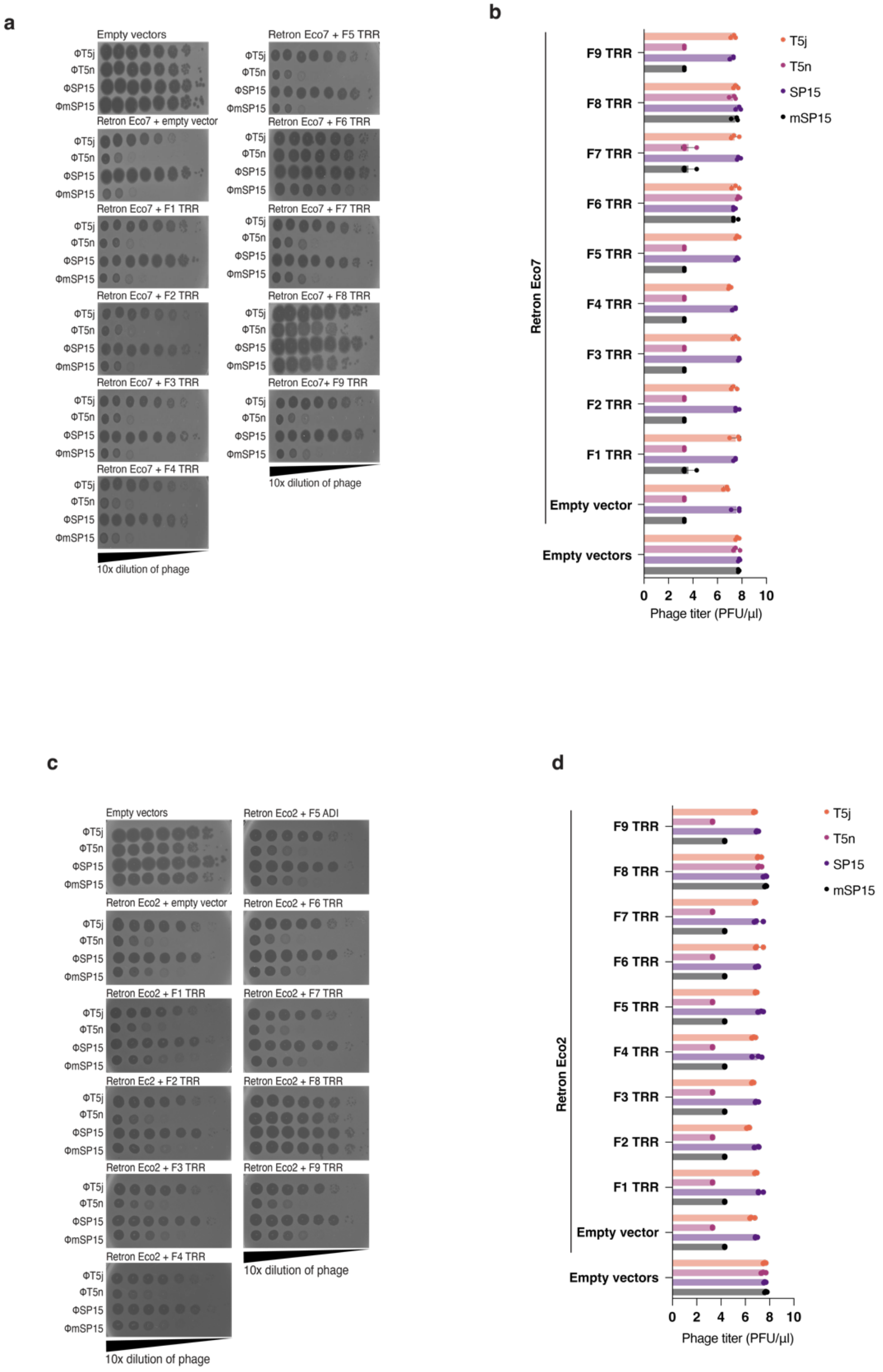

Supplementary Figure 3.

Co-expression of TRR fragment from SP15 and retrons. Co-expression of the TRR fragment from SP15 with retron-Eco7 (A, B) or Eco2 (C, D). Spot assay of wild-type phages (T5j and SP15) and their respective mutants (T5n and SP15m) on bacteria carrying fragmented TRR and retron-Eco7 (A) or Eco2 (C). (B) Quantified phage titer propagated in bacteria carrying different TRR and retron-Eco7 (B) or Eco2 (D). The experiments were performed in three biological replicates. Data are presented as mean values ± SD. Source data are provided as a Source Data File.

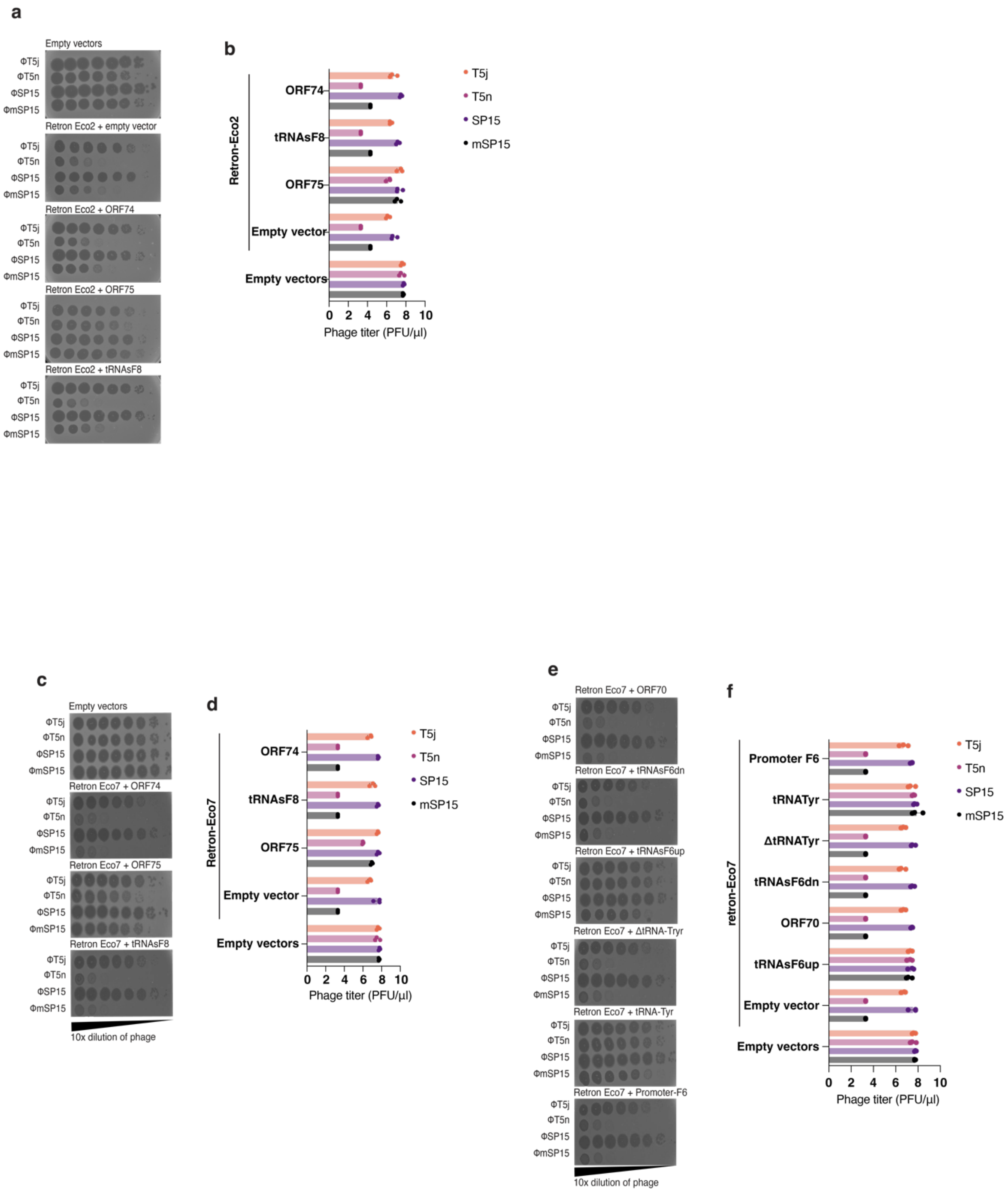

Supplementary Figure 4.

Fragmentation of the TRR fragments 6 and 8. Co-expression of the TRR fragment 6 (F6) or 8 (F8) with retron-Eco2 (A, B) or Eco7 (C–F). Spot assay of wild-type phages (T5j and SP15) and their respective mutants (T5n and SP15m) on bacteria co-expressing genetic component of F8 and retron-Eco2 (A) or Eco7 (C). Quantified phage titer propagated in bacteria carrying genetic component of F8 and retron-Eco2 (B) or Eco7 (D). Spot assay (E) and quantified phage titer (F) (wild-type and mutants) on bacteria co-expressing genetic components of F6 and retron-Eco7. The experiments were performed in three biological replicates. Data are presented as mean values ± SD. Source data are provided as a Source Data File.

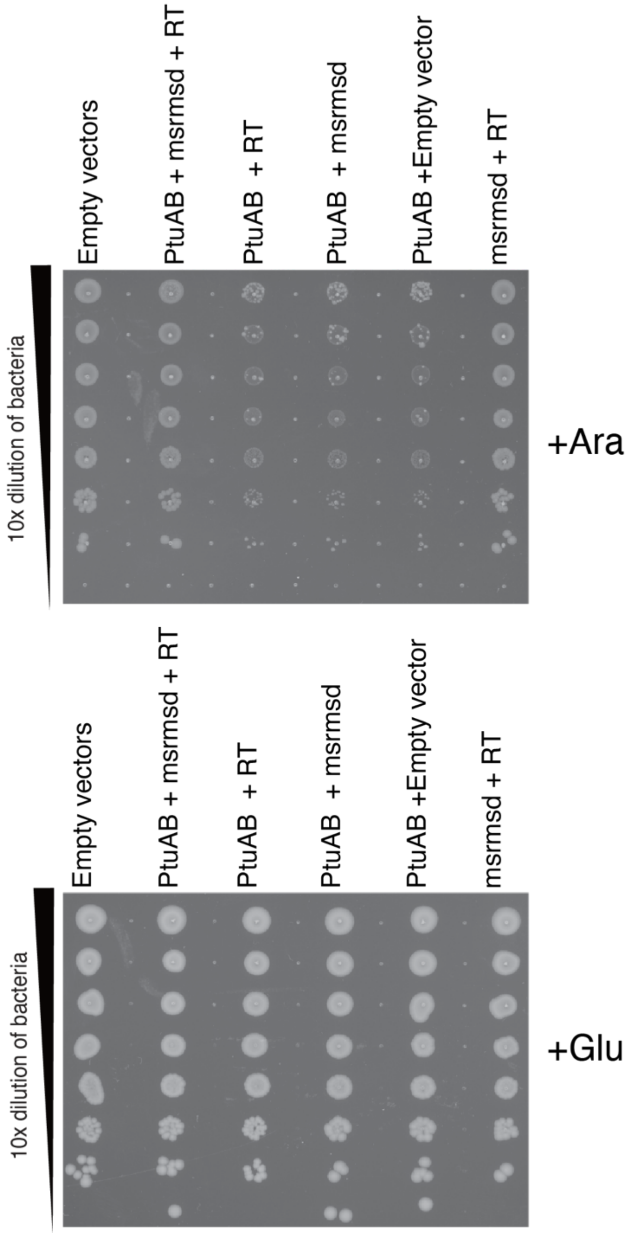

Supplementary Figure 5.

Toxicity assay of bacteria co-expressing the retron-Eco7 component in two inducible plasmids. The toxin component, PtuAB, was expressed under the pBAD inducible promoter, whereas the anti-toxin component RT/msrmsd/both RT and msrmsd were expressed under the pATc inducible promoter. The anti-toxin was continuously expressed by adding 50 ng/mL of anhydrous tetracycline in both induced toxin conditions (Arabinose added) (top) or non-induced toxin conditions (Glucose added) (bottom).

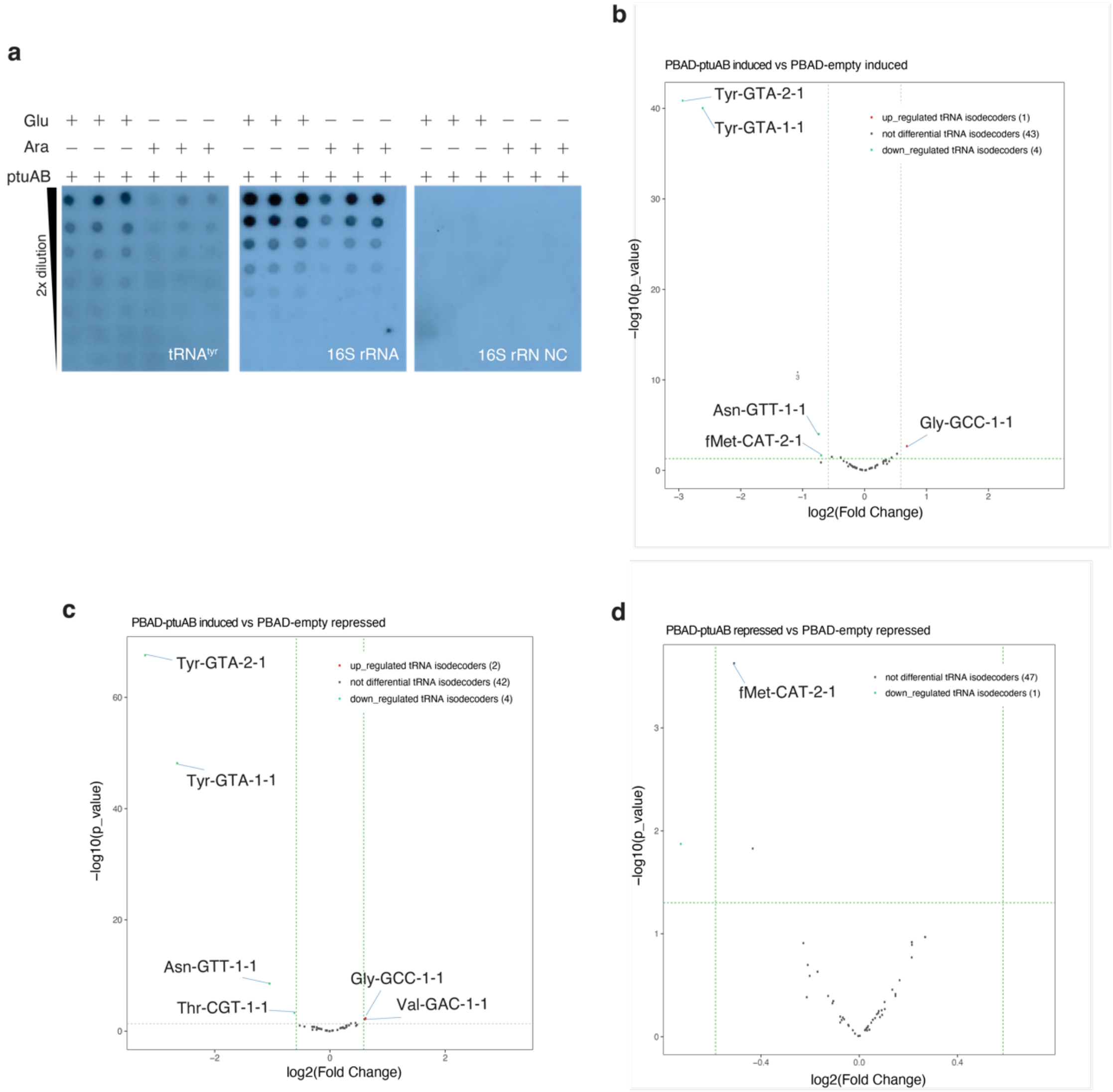

Supplementary Figure 6.

The expression of PtuAB from Ec78 resulted in the degradation of tRNA-Tyr. (A) RNA hybridization dot blot assay of bacteria that express PtuAB. From left to right; tRNA-Tyr, 16S rRNA, and 16S rRNA negative control. Sense oligo of 16S rRNA was used as the negative control. Expression level comparison between bacteria with induced PtuAB and induced empty vector (B); with induced PtuAB and repressed empty vector (C); and with repressed PtuAB and repressed empty vector (D). Induction and repression of PtuAB expression were performed with 0.2% of arabinose and glucose, respectively. Experiments for results in (B), (C), and (D) were performed in duplicates.

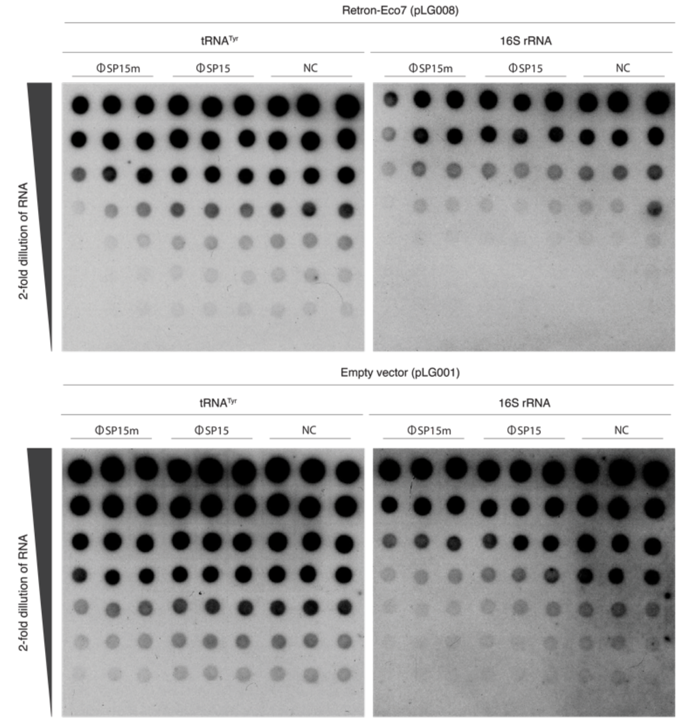

Supplementary Figure 7.
RNA hybridization dot blot assay of bacteria infected with phage SP15 or SP15m. The top panel shows bacteria carrying retron-Eco7, while the bottom panel shows bacteria carrying an empty vector. Negative control (NC) refers to bacteria without phage infection. Experiments were performed in triplicate.

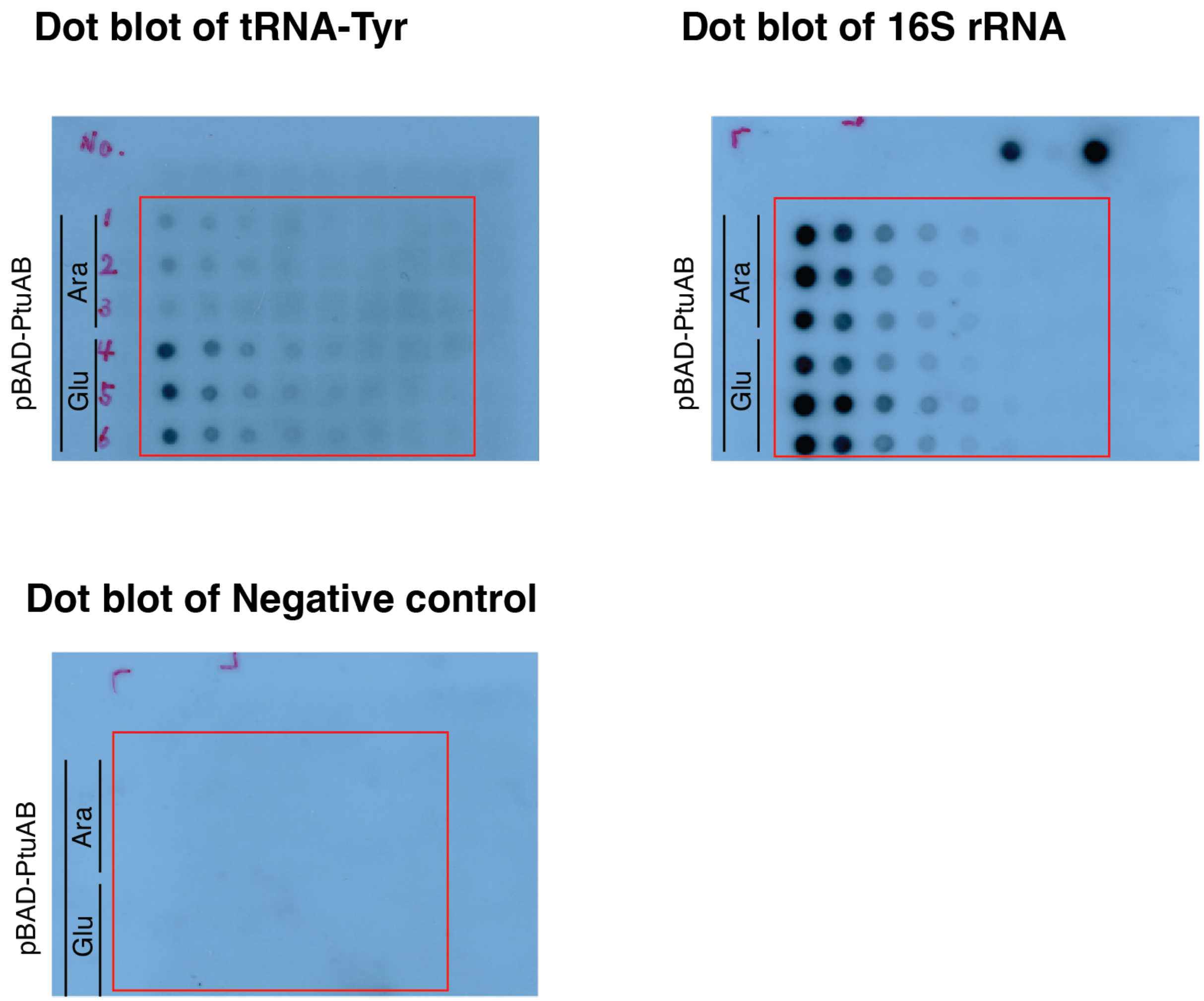

Supplementary Figure 8.

Uncropped photo of RNA hybridization dot blot from Supplementary Figure 6.

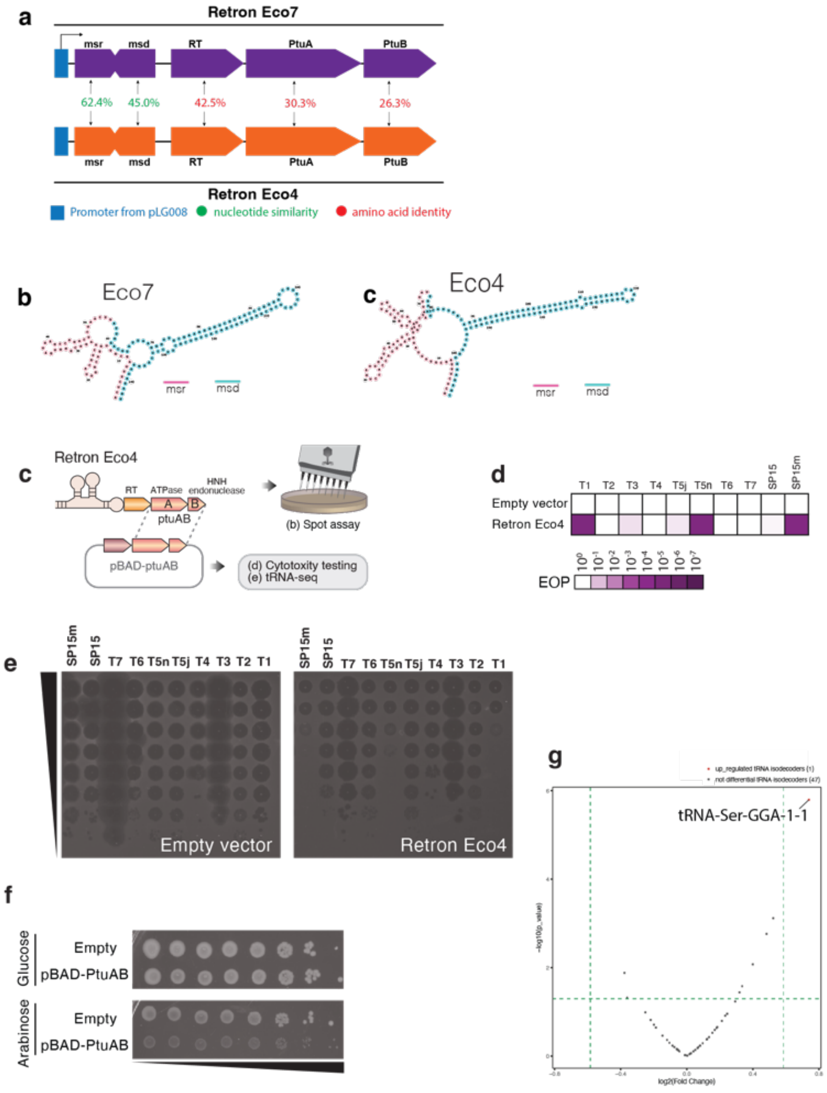

Supplementary Figure 9.

Characterization of retron-Eco4. (A) Genomic alignment between retron-Eco7 and retron-Eco4. (B) Predicted RNA structure of retron-Eco7 and -Eco4 based on RNAfold prediction[4]. (C) Figure illustrating expression of PtuAB from retron-Eco8 under the pBAD inducible plasmid. Bacteria carrying pBAD-PtuAB were used for cytotoxicity assay and tRNA-sequencing. Defense activity of retron-Eco4 on various phages shown in a heatmap (D) and spot assay (E). Source data are provided as a Source Data File. (F) Bacterial growth arrest observed following overexpression of PtuAB. (G) Volcano plot illustrating the results of tRNA-sequencing in bacteria expressing PtuAB. The fold-change was determined by comparing the total tRNA expression observed in bacteria carrying pBAD-PtuAB induced with arabinose to the tRNA expression observed in bacteria carrying pBAD-PtuAB repressed with glucose.

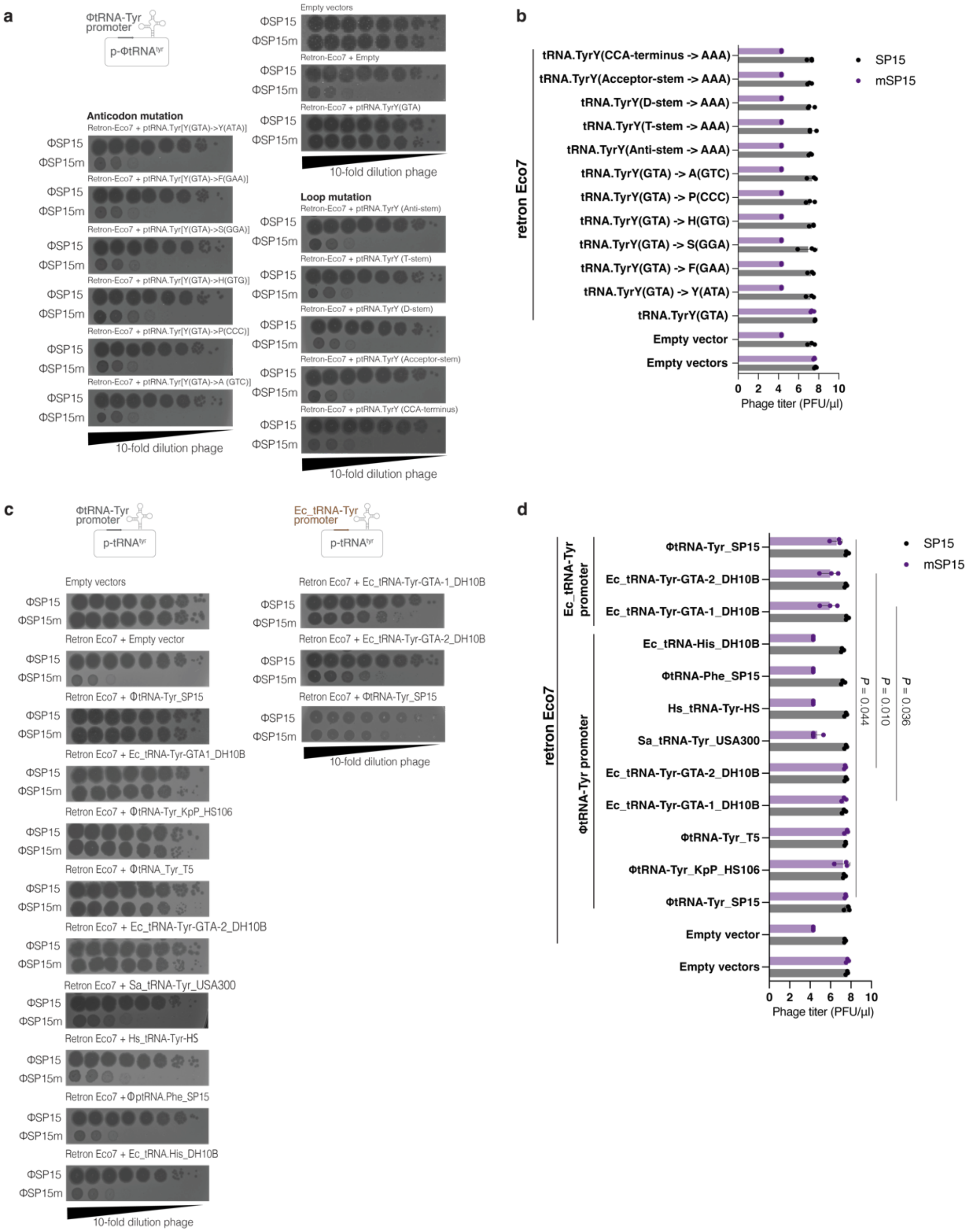

Supplementary Figure 10. Co-expression of retron-Eco7 and tRNA-Tyr.

(A) Spot assay and (B) quantified phage titers (SP15 and SP15m) on bacteria carrying retron-Eco7 and various tRNA-Tyr_SP15 mutants. (C) Spot assay and (D) quantified phage titers (SP15 and SP15m) on bacteria carrying retron-Eco7 and various tRNAs from different organisms. For tRNA-Tyr from E. coli (Ec_tRNA-Tyr-GTA-2_DH10B and Ec_tRNA-Tyr-GTA-1_DH10B) and phage SP15 (ΦtRNA-Tyr_SP15), two different promoters, either the *E. coli* tRNA promoter or the phage tRNA promoter, were used. The experiments were performed in three biological replicates. Data are presented as mean values ± SD. Statistical significance is indicated by the *P*-value in the graph. Statistical analysis was performed using a two-tailed Student's t-test, assuming equal variances. Source data are provided in the Source Data file.

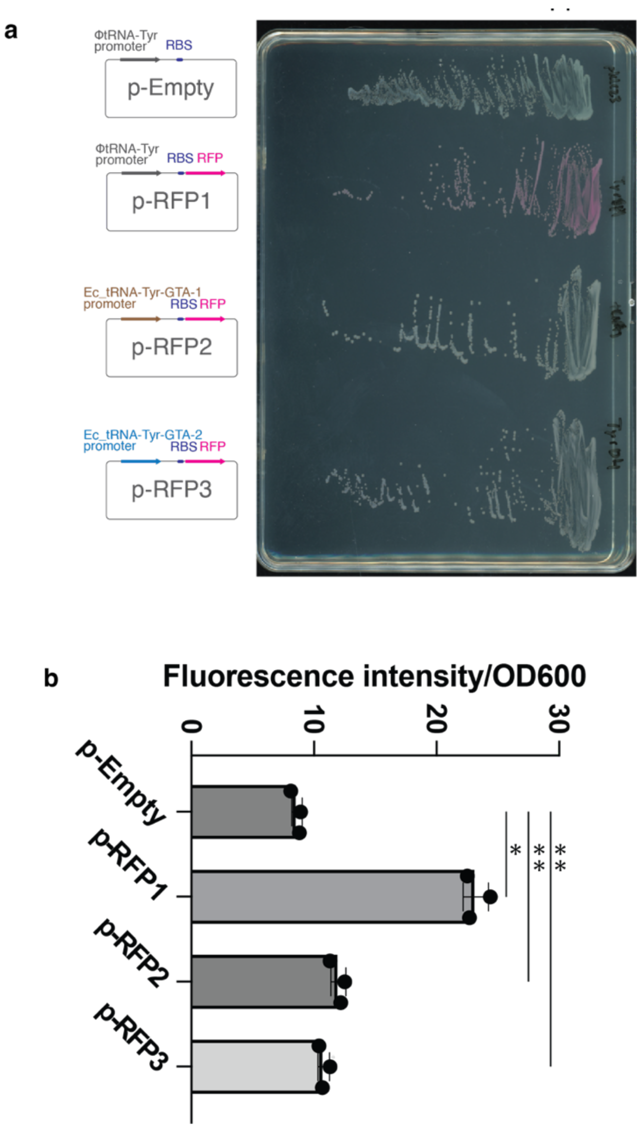

Supplementary Figure 11.

Evaluating the activity of tRNA promoter described in Fig 4F. (A) Promoter activity was evaluated by expressing red fluorescence protein (RFP). From top to bottom; empty vector (p-Empty) consisting of tRNA promoter from SP15 and ribosome-binding site (RBS), plasmid p-RFP1 consisting of tRNA promoter from SP15, RBS, and RFP, plasmid p-RFP2 consisting of tRNA promoter from *E coli* tRNA-Tyr-GTA-1, RBS, and RFP, plasmid p-RFP3 consisting of tRNA promoter from *E coli* tRNA-Tyr-GTA-2, RBS, and RFP. (B) Fluorescence intensity of bacteria expressing RFP under different tRNA promoters.

PrrC toxin

```
                                                             Walker A
EcoPrrC             MGKTLSEIAQQLSTPQKVKKTVHKEVEAIRAVPKVQLIYAFNGTGKTRLSRDFKQLLESK
PrrC170             -------MANKLATFQELGEIAAHLREKLEDK-KYVLLFAYNGTGKTRLSMEFKELGKNG
                           :*::*:* *:: : . :  * :.   *  *::*:********* :**:* :.

                                               PrrC box
EcoPrrC             VHDGEGEDEAEQSALSRKKILYYNAFTEDLFYWDNDLQEDAEPKLKVQPNSYTNWLLTLL
PrrC170             DD---------------RDTLYFNAFTEDLFNWDNDLEHDSKRVLRLNRESR---FFDGL
                     .               :. **:******** *****:.*::  *::: :*    ::  *

                                                                    C-loop
EcoPrrC             KDLGQDSNIVRYFQRYANDKLTPHFNPDFTEITFSMERGNDERSAHIKLSKGEESNFIWS
PrrC170             EELEMENRIAPILARYADFNFYIDYT--DWEIIFFRETDSEGDPIPIKVSRGEENIFIWC
                    ::*  :..*.  : ***: ::  .:.    ** *  * ..:  .  **:*:***. ***.

                                                  Walker B        D-loop
EcoPrrC             VFYTLLDQVVTILNVADPDARETHAFDQLKYVFIDDPVSSLDDNHLIELAVNLAGLIKSS
PrrC170             FFLVVARLALDSE--------EGAVYSWVKFIYIDDPISSLDDNNAVAVAHHLAQLFKTS
                    .* .:   .:           *  .:. :*:::****:******: : :* :** *:*:*

                           H-loop
EcoPrrC             ESDLKFIITTHSPIFYNVLFNELNGKVCYMLESFEDG-TFALTEKYGDSNKSFSYHLHLK
PrrC170             RNDIKVTLSSHHTLFFNVMCNEWGNAVKYFLGKNEGGNGYTLKPMYGDTAR--FYHVAML
                    ..*:*. :::* .:*:**: ** .. * *:* . *.*  ::*.  ***: :   **: :

EcoPrrC             QTIEQAIADNNVERYHFTLLRNLYEKTASFLGYPKWSELLPD----DKQLYLSRIINFTS
PrrC170             RDLKKAADSGELYTYHFNILRNLMEKTATFHGYTDFSHFISVENDDDEKTLLTRLVQVLN
                    : :::*  ..::  ***.:**** ****:* **..:*.::.     *::  *:*:::. .

EcoPrrC             HSTLSNEAVAEPTPAEKATVKLLLDHLKNNCGFWQQEQKNG
PrrC170             HGGYSLLEPIEMIPENKSHFRKILNDFLKTYSFNQGIFS--
                    *.  *     *  * :*: .: :*:.: :. .* *   .
```

Supplementary Figure 12. Amino acid alignment between the PrrC toxins of PrrC170 and EcoPrrC. Conserved ABC-ATPase domain and PrrC domain are highlighted in red letters.

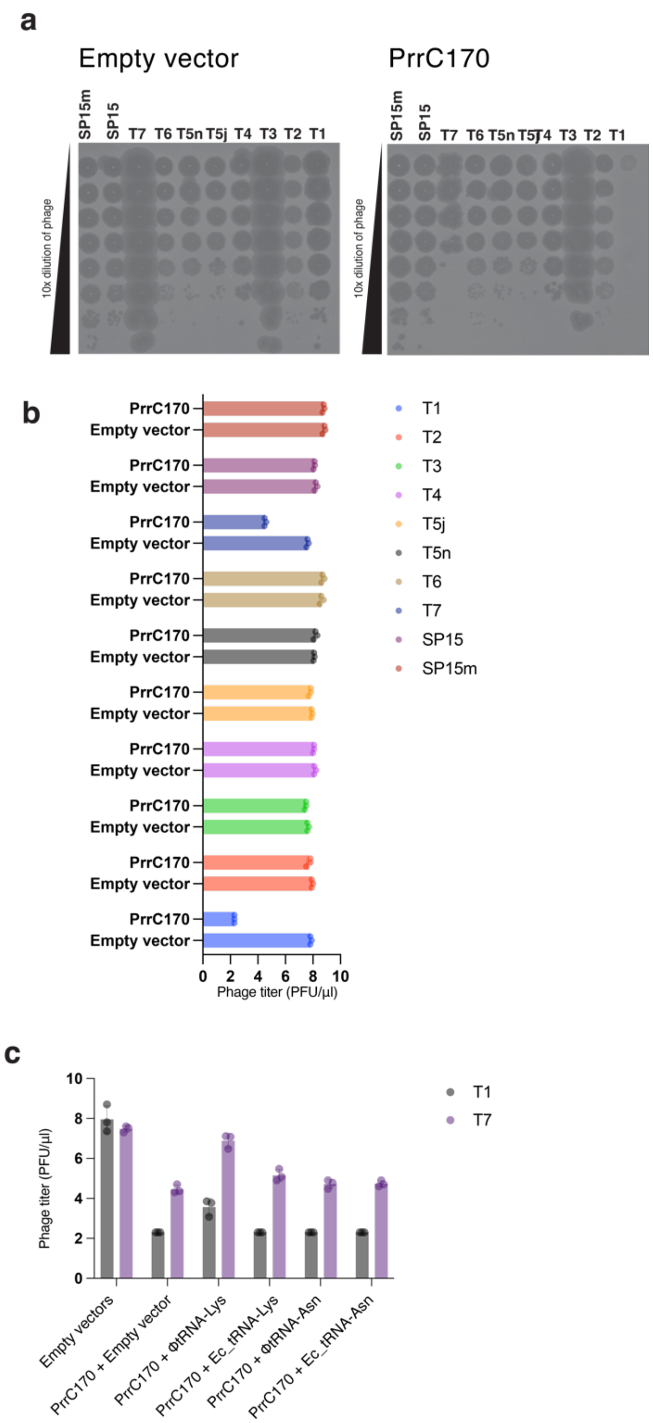

Supplementary Figure 13. Defense activity of the PrrC170 system. Spot assay (A) and quantified phage titer (B) of various phages on bacteria carrying PrrC170. (C) Quantified phage titer of T1 and T7 on bacteria carrying PrrC170 and tRNA. *E. coli* tRNA^{Lys} (Ec_tRNA-Lys), *E. coli* tRNA^{Asn} (Ec_tRNA-Asn), tRNA^{Lys} from phage SP15 (ΦtRNA-Lys), and tRNA^{Asn} from phage SP15 (ΦtRNA-Asn), were used. The experiments were performed in three biological replicates. Data are presented as mean values ± SD. Source data are provided as a Source Data File.

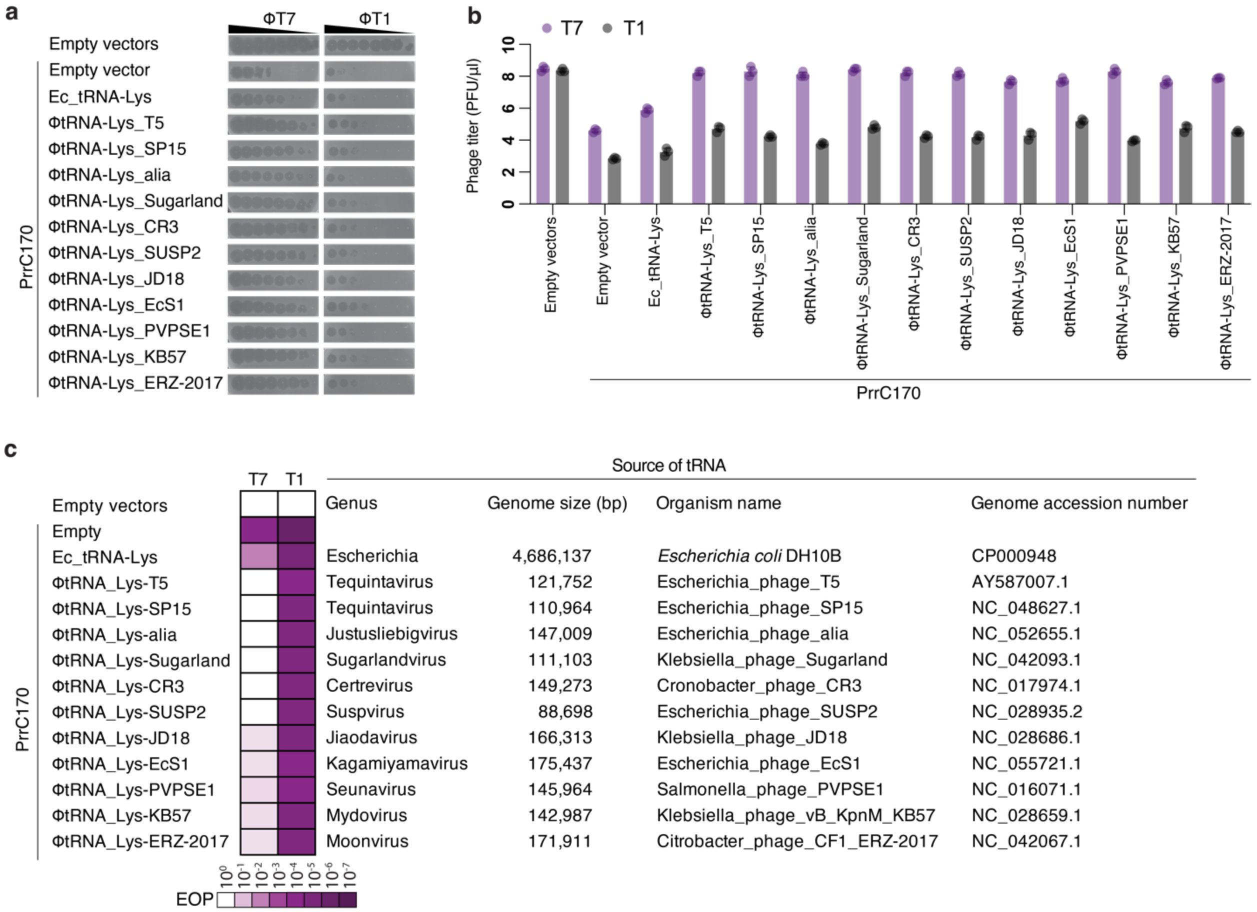

Supplementary Figure 14. Co-expression of PrrC170 and tRNA-Lys from *E. coli* and various phages. Spot assay (A) and quantified phage titer (B) for T1 and T7 phages on bacteria expressing PrrC170 and different tRNA-Lys. Heatmap (C) shows the changes in the efficiency of plating (EOP) in the phage assay on bacteria expressing PrrC170 and various tRNA-Lys. The sources of tRNA-Lys used in the assay are indicated. The experiments were performed in three biological replicates. Data are presented as mean values ± SD. Source data are provided as a Source Data File.

Supplementary References

1. Gilchrist, C. L. M. & Chooi, Y.-H. clinker & clustermap.js: automatic generation of gene cluster comparison figures. *Bioinformatics* **37**, 2473–2475 (2021).
2. Oerum, S. *et al.* Structures of B. subtilis Maturation RNases Captured on 50S Ribosome with Pre-rRNAs. *Mol Cell* **80**, 227-236.e5 (2020).
3. Kiga, K. *et al.* Development of CRISPR-Cas13a-based antimicrobials capable of sequence-specific killing of target bacteria. *Nat Commun* **11**, (2020).
4. Gruber, A. R., Lorenz, R., Bernhart, S. H., Neubock, R. & Hofacker, I. L. The Vienna RNA Websuite. *Nucleic Acids Res* **36**, W70–W74 (2008).
