# Supplementary material for: Evasion of antiviral bacterial immunity by phage tRNAs
Source: Nat Commun. 2024 Nov 11;15:9586. doi: 10.1038/s41467-024-53789-y (PMC11555353; doi:10.1038/s41467-024-53789-y)
Supplement: Supplementary file 2 — Description of Additional Supplementary Files [file 41467_2024_53789_MOESM2_ESM.pdf]

Description of Additional Supplementary Files:

Supplementary Data 1: Predicted tRNAs in the TRR region of T5-like phages. (a) Number of tRNA in the TRR region of each T5-like phage. (b) Number of T5-like phages and their corresponding tRNAs.

Supplementary Data 2: tRNA count of PtuAB Retron-Eco7 induced/non-induced bacteria.

Supplementary Data 3: tRNA count of PtuAB retron-Ec83 (Eco4) induced/noninduced bacteria.

Supplementary Data 4: tRNA count of PrrC induced/non-induced bacteria.

Supplementary Data 5: Prediction of antidefense gene in TRR region of T5-like phages.

Supplementary Data 6: Plasmids used in this study.

Supplementary Data 7: Bacteria and bacteriophages used in this study.

Supplementary Data 8: Primers used in this study.

Supplementary Data 9: Synthetic plasmids and oligo DNA of tRNAs used in this study.

(A) List of synthetic plasmids used in the study.

(B) Oligo DNA sequences of tRNAs used in the study
